# Physical activity is related to disease severity and fatigue, but not to relapse rate in persons with relapsing remitting multiple sclerosis – a self-reported questionnaire based study

**DOI:** 10.3389/fneur.2023.1217000

**Published:** 2023-07-31

**Authors:** Marit L. Schlagheck, Sven T. Hübner, Niklas Joisten, David Walzik, Annette Rademacher, Florian Wolf, Jens Bansi, Clemens Warnke, Philipp Zimmer

**Affiliations:** ^1^Division of Performance and Health, Institute for Sport and Sport Science, Technical University Dortmund, Dortmund, Germany; ^2^Department for Molecular and Cellular Sports Medicine, Institute for Cardiovascular Research and Sports Medicine, German Sport University Cologne, Cologne, Germany; ^3^Marianne-Strauß-Klinik, Behandlungszentrum Kempfenhausen für Multiple Sklerose Kranke gGmbH, Berg, Germany; ^4^Neurological Rehabilitation Centre Godeshöhe, Bonn, Germany; ^5^Department of Research and Development, Kliniken Valens, Valens, Switzerland; ^6^Department of Health, OST – Eastern Swiss University of Applied Sciences, St. Gallen, Switzerland; ^7^Department of Neurology, Faculty of Medicine and University Hospital Cologne, University of Cologne, Cologne, Germany

**Keywords:** multiple sclerosis, physical activity, disease severity, fatigue, relapsing remitting multiple sclerosis

## Abstract

**Introduction:**

Based on theoretical models, physical activity has been introduced as a promoting method to mitigate the disease severity, fatigue and relapse rate in multiple sclerosis. The primary objective of the study was to investigate the relation between self-reported physical activity level and disease severity, fatigue and relapse rate in persons with relapsing remitting multiple sclerosis (RRMS).

**Methods:**

A survey was offered to persons with RRMS from March 2019 to August 2021 (*n* = 253). Physical activity level, fatigue and disease severity were determined using the Godin Leisure-Time Questionnaire (GLTEQ), the Patient Determined Disease Steps (PDDS) scale and the Fatigue Scale for Motor and Cognitive Functions (FSMC). Additionally, participants’ relapse rate was recorded.

**Results:**

Bivariate correlations revealed an inverse relation between physical activity level and PDDS (*ρ* = −0.279; *p* < 0.001) as well as between physical activity and FSMC (*r* = −0.213, *p* < 0.001), but not between physical activity and relapse rate (*r* = 0.033, *p* > 0.05). Multiple linear regression analyses explained 12.6% and 5.2% of the variance of PDDS and FSMC.

**Conclusion:**

Our findings confirm a relation between self-reported physical activity, disease severity and fatigue in persons with RRMS. However, self-reported physical activity level does not seem to affect the annualised relapse rate.

## 1. Introduction

Multiple sclerosis (MS) is a chronic immune-mediated disease. Persons with MS (pwMS) are likely to experience complex disabilities, including a decline in physical and cognitive function, as well as progressive depression and fatigue (1). A growing body of literature confirms positive effects of regular physical activity and exercise on disease-specific symptoms of MS (2–4). Recently, a comprehensive non-systematic review summarised the role of physical activity and exercise as tertiary (i.e., reducing symptoms appearance), secondary (i.e., provoking disease-modification), and even primary (i.e., reducing the risk of developing MS) prevention method of MS (5). The authors introduced a theory-based model that shows physical activity to reduce inflammatory disease activity and progression in pwMS (*exercise-induced postponement theory*). This theoretical framework is subject by preclinical animal models showing adaptations at the cellular level, such as attenuation of cellular infiltration and inhibition of pro-inflammatory mediators in the central nervous system (6, 7). Although an increasing number of exercise intervention studies focuses on the effects of acute physical activity on symptoms such as fatigue and cognition or motor impairments in pwMS only a few investigations with heterogeneous study quality have been conducted so far assessing disease-modifying effects (8). Even less is known about the disease-modifying effects of regular lifestyle physical activity, which also covers unplanned and unstructured physical movements in daily life (5, 9).

With this background, the objective of the current study was to investigate a potential relation between physical activity and disease severity, fatigue and relapse rate in persons with relapsing remitting multiple sclerosis (RRMS).

## 2. Materials and methods

This cross-sectional study was approved by the ethics committee of the German Sport University Cologne (028/2019), registered in the German Clinical Trials Register (DRKS00016624) and was performed according to the latest Declaration of Helsinki. All participants provided written informed consent, and data was collected anonymized.

### 2.1. Recruitment

From March 2019 to August 2021, 253 people with RRMS were recruited through the homepages of the German Sport University Cologne, Germany and the German Multiple Sclerosis Society (North-Rhine-Westphalia state association) and through clinics in Germany and Switzerland (Neurological Rehabilitation Centre Godeshöhe, Germany and Clinic of Valens, Switzerland) to fill out an online survey hosted by the Qualtrics software (Qualtrics^®^, Provo, Utah, United States). The survey contained questions about (1) sociodemographic data, (2) physical activity level (3) disease severity, (4) fatigue and (5) number of relapses. Inclusion criteria for this analysis comprised a definite RRMS diagnosis, being at least 18 years of age and being a German-native speaker. There were no exclusion criteria once the inclusion criteria were met.

### 2.2. Measurements

Sociodemographic data were collected via several multiple-choice questions and classified as described. The participants’ residence was classified as Germany, Switzerland or other. Their sex was classified as male, female or divers. The participants’ highest educational level was sectioned into five categories: “Hauptschule” (9 years), “Realschule” (10 years), “Abitur” (12–13 years), an occupational certificate, or university degree. Furthermore, age, height and weight were reported and the body mass index (BMI) was calculated.

Physical activity level was assessed using the Godin Leisure-Time Questionnaire (GLTEQ) (10). In this self-evaluation report the frequency of strenuous (heart beats rapidly, e.g., running), moderate (e.g., not exhausting, e.g., fast walking), and mild (minimal effort, e.g., easy walking) physical activity bouts lasting for more than 15 min during a typical 7-day period is measured. The GLTEQ has been described as a valid and appropriate self-report instrument and is commonly applied in research among pwMS (11). To evaluate the effect of health-promoting physical activity, the health contribution score (HCS) was computed as the sum of the strenuous activity bouts * 9 and moderate activity bouts * 5 (12). The HCS allows an interpretation of the physical activity level with respect to the public-health guidelines (13, 14) and recommendations for pwMS (15, 16).

The disease severity was assessed via the Patient Determined Disease Steps (PDDS) scale (17). The PDDS is a patient-reported outcome comprising a scale from 0 (normal/no restrictions of activities due to MS-specific symptoms) to 8 (bedridden). It provides a validated and easily applicable alternative to the clinician-administered Expanded Disability Status Scale, to which the scores correlate highly (*ρ* = 0.783) (18).

Fatigue was assessed via the Fatigue Scale for Motor and Cognitive Functions (FSMC) (19). The questionnaire contains 20 statements regarding fatigue-related restrictions in daily life, which are rated on a five-point Likert Scale. The sum score shows the extent of persisting fatigue in daily life, with higher values representing a greater severity of the symptoms. Additionally, two subscales offer the possibility to differentiate between motor fatigue and cognitive fatigue.

Furthermore, the participants were asked to report their total number of relapses. The annualised relapse rate was calculated by dividing the total number of relapses by year of MS duration. Including patients with a disease duration of less than 2 years led to an overestimation of their relapse rate, thus not being comparable to others (and subsequently to highly skewed and kurtosed data). Moreover, the relapse rate may not reflect disease severity in patients with extended disease duration (> 10 years) as the number of relapses decreases over time (20). Thus, the annualised relapse rate was included in further analyses only for those participants who had a defined MS diagnosis for at least 2 and a maximum of 10 years.

### 2.3. Statistical analyses

Statistical analyses were conducted using SPSS version 28.0 (IBM, Armonk, NY, United States) and graphics were done using R, version 4.1.1. Data were checked for linearity (via quantile-quantile plots), skewness and kurtosis. The significance level was set as *p* ≤ 0.050. For FSMC sum score and FSMC subscales, the significance level was set as *p* ≤ 0.017 according to Bonferroni alpha correction for multiple testing.

Bivariate correlations (Pearson *r* for continuous variables or Spearman’s rho *ρ* for ordinal variables) were conducted to determine potential relation between physical activity level (i.e., GLTEQ-HCS), disease severity (i.e., PDDS), fatigue (i.e., FSMC sum score and FSMC subscales) and/or the annualised relapse rate.

Thereafter, four multiple linear regression models were conducted to observe the extent of correlations between disease-related outcomes (i.e., PDDS, FSMC sum score, FSMC motor subscale, and FSMC cognition subscale) and physical activity behaviour (i.e., GLTEQ-HCS as first predictor) as well as participants’ characteristics (i.e., sex, age, BMI, MS duration, application of disease-modifying therapy as further predictors). Predictors were chosen based on theoretical considerations and previous calculations. Data met the following assumptions: the independency of residuals by Durbin Watson Test (1 < *x* < 3) (21) lack of multicollinearity (tolerance statistics > 0.2, and variance inflation factor values < 2) (21) and homoscedasticity (visually via histogram of studentised residuals).

## 3. Results

### 3.1. Participants’ characteristics

Sociodemographic and clinical characteristics of all participants included in the calculations are displayed in Table 1. Eighty-four percent of participants were female, representing a slightly greater percentage than in a typical distribution for pwMS (22). Overall, participants were highly educated and characterised by mild to moderate disability (PDDS range 0–6, mean 1.5 ± 1.3). At the time of study participation, 41.9% of participants did not meet the public-health recommendations for physical activity (i.e., GLTEQ-HCS < 24) (13, 14).

**Table 1:** Participant’s characteristics (*n* = 253).

Categorial variables	*n*	%
Residence		
Germany	225	88.9
Switzerland	14	5.5
Other	14	5.5
Sex		
Female	212	83.8
Male	41	16.2
Smoking behaviour		
Smoker	33	13.0
Non-smoker	220	87.0
Educational level^a^		
Hauptschule	2	0.8
Realschule	23	9.2
University entrance qualification	23	9.2
Occupational certificate	79	31.6
University degree	123	49.2
Disease-modifying therapy		
Yes	197	77.9
No	46	22.1

Continuous variables	Mean ± SD	95% CI
Age (years)	43.5 ± 11.0	42.1–44.8
BMI (kg/m^2^)	24.6 ± 4.7	24.0–25.2
MS duration (years)	9.7 ± 7.9	8.7–10.7
Age at diagnosis (years)	33.8 ± 10.5	32.6–35.2
Annualised relapse rate^b^	0.8 ± 0.6	0.7–0.9
PDDS score	1.5 ± 1.3	1.4–1.7
FSMC sum score	62.2 ± 21.0	59.6–64.8
FSMC motor score	31.7 ± 10.5	30.4–33.0
FSMC cognition score	30.5 ± 11.2	29.1–31.8
GLTEQ-HCS	37.0 ± 24.6	34.0–40.1

Data is presented as mean ± standard deviation (SD) and with 95%-confidence interval (CI) for continuous variables and in percentages (%) for distributions; *n* study population; BMI, body mass index; PDDS, Patient Determined Disability Status Scale; FSMC, Fatigue Scale for Motor and Cognitive Functions; GLTEQ-HCS, Godin Leisure Time Questionnaire-health contribution score.

a Data missing for three participants.

b Data available only for those participants who had a defined MS diagnosis for at least 2 and maximum 10 years, *n* = 114.

### 3.2. Biariate correlations

The relation of GLTEQ-HCS and PDDS, FSMC sum score, FSMC subscales, annualised relapse rate and potential confounding variables are shown in Table 2. There were significant relations between the GLTEQ-HCS and PDDS, all FSMC scores, age, and BMI. GLTEQ-HCS did not significantly correlate to the annualised relapse rate (*p* > 0.05).

**Table 2:** Bivariate correlations.

	PDDS 0-6		PDDS 0, 1, 2	
	*n*	GLTEQ-HCS	*n*	GLTEQ-HCS
Age	253	–0.134*	195	–0.070
BMI	253	–0.223***	195	–0.176*
PDDS^a^	253	–0.279***	195	–0.112
Annualised relapse rate^b^	114	0.033	92	0.098
FSMC total score	253	–0.213***	195	–0.190**
FSMC motor score	253	–0.220***	195	–0.183*
FSMC cognition score	253	–0.192**	195	–0.186**

Pearson product–moment correlations have been conducted if not reported otherwise; *n* study population; GLTEQ-HCS, Godin Leisure Time Exercise-health contribution score; MS, multiple sclerosis; PDDS, Patient Determined Disease Steps; FSMC, Fatigue Scale for Motor and Cognition. *p ≤ 0.05; **p ≤ 0.01; ***p ≤ 0.001.

a Spearman correlation has been conducted.

b Data available only for those participants who had a defined MS diagnosis for at least 2 and maximum 10 years.

When including only ambulatory pwMS (i.e., PDDS 0-2; *n* = 195) there is no significant correlation between PDDS and GLTEQ-HCT (*p* = 0.120).

### 3.3. Multiple regression models

Based on theoretical models and previous correlations, multiple linear regression models were conducted for PDDS, FSMC sum score and FSMC subscales to evaluate the variance explained by the physical activity behaviour and demographic variables.

**Figure 1:**
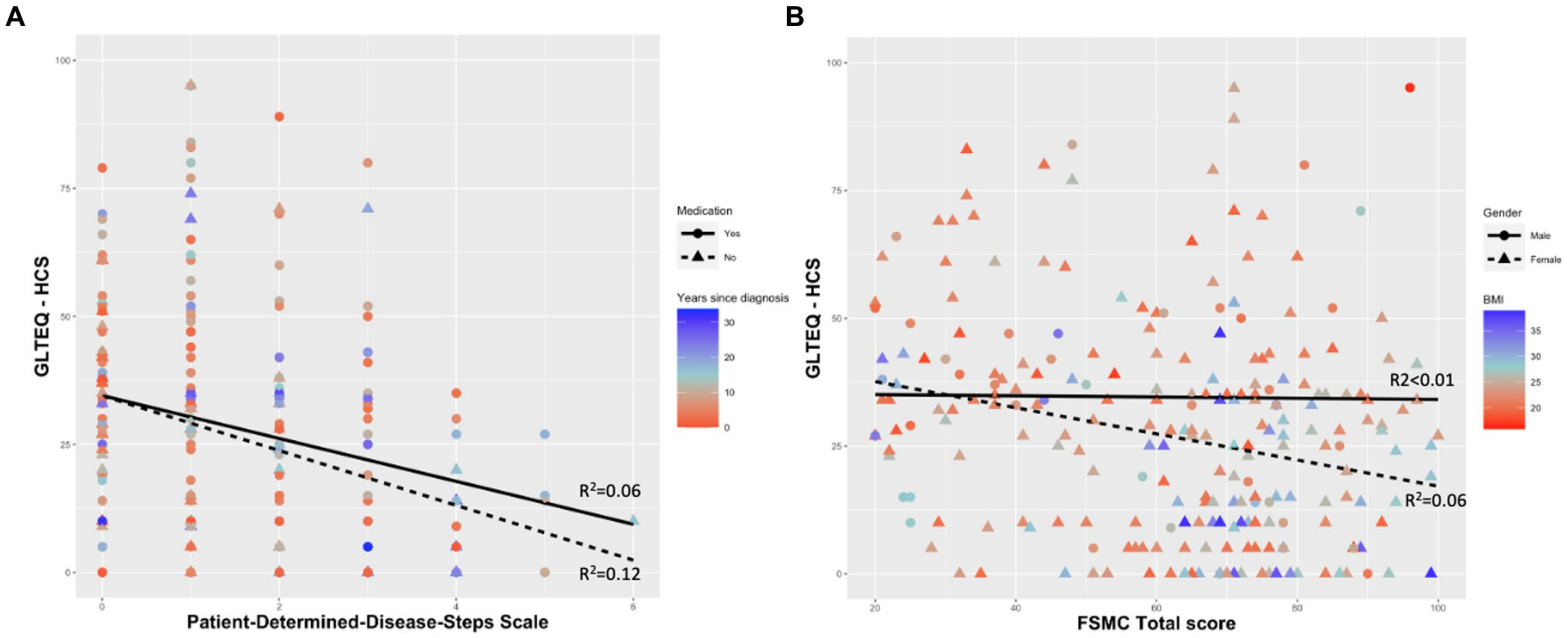
Self-reported physical activity behaviour as a significant predictor for **(A)** Patient-Determined-Disease-Steps Scale and **(B)** Fatigue Scale for Motor and Cognitive Functions. GLTEQ-HCS, Godin Leisure-Time Exercise Questionnaire-health contribution score; FSMC, Fatigue Scale for Motor and Cognitive Functions; BMI, body mass index.

All models showed significant effects of the physical activity behaviour (measured as GLTEQ-HCS). The model including PDDS as dependent variable accounted for 12.6% [*F* (6,246) = 7.032, *p* < 0.001] of the variance. The models including FSMC sum score, FSMC motor score and FSMC cognition score accounted for 5.2% [F (6,246) = 3.318, *p* = 0.004], 5.2% [F (6,246) = 3.325, *p* = 0.004], and 4.5% [F (6,246) = 2.972, *p* = 0.008] of the variance (Table 3). More detailed, higher self-reported physical activity levels (ß = −0.228, *p* < 0.001) were related with a lower physical disability (measured via PDDS). Participants who were disease-modifying medication tended to be characterised by a lower PDDS (*p* = 0.055, Figure 1A). Regarding the outcome fatigue, a higher self-reported physical activity level was related with a significantly lower FSMC sum score (ß = −0.175, *p* = 0.007) (Figure 1B), FSMC motor score (ß = −0.191, *p* = 0.003) and FSMC cognition score (ß = −0.148, *p* = 0.023). Being female was related with a higher FSMC motor score (ß = 0.125, *p* = 0.048) and higher BMI was related with a higher FSMC cognition score (ß = 0.136, *p* = 0.038).

**Table 3:** Relationship between physical activity level and PDDS and components of fatigue.

	*B*	SE	*ß*	*p*	*B*	SE	*ß*	*p*
	PDDS				FSMC total score			
Constant	0.621	0.781		0.427	47.528***	12.767		< 0.001
GLTEQ-HCS	0.014***	0.004	−0.228	< 0.001	−0.170**	0.062	−0.175	0.007
Age	0.009	0.008	0.074	0.263	−0.055	0.131	−0.029	0.677
BMI	0.029	0.018	0.102	0.103	0.496	0.292	0.110	0.091
Sex^a^	0.156	0.218	0.043	0.474	6.973	3.563	0.123	0.051
MS duration	0.036**	0.011	0.211	0.002	0.029	0.186	0.011	0.878
Medication intake^b^	−0.368	0.191	−0.115	0.055	−2.900	3.124	−0.058	0.354
*R*^2^	0.146				0.075			
Adjusted *R*^2^	0.126				0.052			
F statistic (df = 6, 246)	7.032***			<0.001	3.318**			0.004
	FSMC motor scale				FSMC cognition scale			
Constant	27.384***	6.419		<0.001	20.143**	6.843		0.004
GLTEQ-HCS	−0.093**	0.031	−0.191	0.003	−0.077*	0.033	−0.148	0.023
Age	−0.041	0.066	−0.043	0.531	−0.013	0.070	−0.013	0.850
BMI	0.169	0.147	0.075	0.250	0.326*	0.157	0.136	0.038
Sex^a^	3.562*	1.791	0.125	0.048	3.411	1.910	0.112	0.075
MS duration	0.025	0.093	0.019	0.787	0.003	0.100	0.002	0.973
Medication intake^b^	−1.823	1.571	−0.072	0.247	−1.077	1.674	−0.040	0.521
*R*^2^	0.075				0.068			
Adjusted *R*^2^	0.052				0.045			
F statistic (df = 6, 246)	3.325**			0.004	2.972**			0.008

PDDS, Patient Determined Disease Steps; FSMC, Fatigue Scale for Motor and Cognition Functions; GLTEQ-HCS, Godin Leisure Time Exercise Questionnaire-health contribution score; BMI, body mass index; *R*^2^, coefficient of determination; df, degrees of freedom; *B*, unstandardised coefficient; SE, standard error; *ß*, standardised coefficient; *p*, significance. *p < 0.050; **p < 0.010; ***p < 0.001.

a Male served as the reference.

b Intake of disease-modifying medication served as the reference (vs. non-intake).

## 4. Discussion

Assessing the relation between physical activity and disease severity, relapse rate and fatigue in pwMS, this study confirms negative relationship between a self-reported physical activity level, disability severity and fatigue. In addition, multiple regression analyses explain 12.6% and 5.2% of the variances in PDDS and FSMC scores, respectively. When all theoretical predictors of disease progression were considered and depending on the measured outcome, MS duration, gender, and BMI were significant whereas current physical activity was the most consistent. It is displayed that increased current physical activity is related with lower disability severity and fatigue. In this regard, our data confirm the proposed *exercise-induced postponement theory* by Dalgas et al. (4). This is in line with results from most cross-sectional (23–26) and longitudinal (27, 28) studies that investigate the relationship between physical activity level and disease progression expressed by symptom exacerbations such as fatigue, or neurological and motor impairment.

On the contrary, our findings do not support a correlation between physical activity and the annualised relapse rate. The rate of relapses represents a common measure to quantify inflammatory disease activity in MS clinical trials (27). In line with our results, Tallner et al., report inconsistent results regarding the relationship between physical activity and the relapse rate (28). Groups categorised according to their physical activity level did not differ significantly for annualised relapse rate. Yet, when these variables were considered in a correlation, a significant inverse relationship emerged (28). It is worth mentioning that the disease duration was not considered in the analyses. However, it is an important parameter as the accuracy of the relapse rate as a prognostic factor has been discussed, especially for long-term disability (20, 27). As the number of relapses decreases over time, its rate seems to be most reliable in the first years of the disease (20). Thus, the results presented by Tallner et al., might not represent the full potentials of physical activity (28). In our investigation, we analysed the relapse rate only for those patients who reported a disease duration of a maximum of 10 years at the time of study participation. We could not compute the relapse rate for patients diagnosed within the last 2 years as it led to an overestimation of their relapse rate. However, the relapse rate might be a more sensible prognostic value for this patient group.

This study has limitations that need to be taken into account when interpreting the results. First, data is restricted to self-reported outcomes, also including disability-scale and number of relapses. Patients with MS might not be aware of their total number of relapses. Future studies might assess both factors using objective measurements such as radiological diagnostic via MRI examination. Furthermore pwMS probably misjudge their level of disability. The PDDS may not be detailed enough and could be supplemented with accelerometer data.

Second, a selection bias may have potentially affected the study sample, as study participants are characterized as highly educates and suffer from a rather benign disease course, which may have impacted the study’s results. Third, the results of our study cannot explain the direction of the relationship between current physical activity level and disease activity or progression. It seems logical that pwMS with a pronounced disability status or fatigue are less capable and/or motivated to perform long-lasting or intense physical activity. Therefore we recalculated the correlation with including only ambulatory pwMS (i.e., PDDS 0-2). After changing the including criteria there is no significant inverse correlation between PDDS (0-2) and GLTEQ-HCT (*p* = 0.12). This confirms that pwMS with a physical impairment are less physical active justified by their impairment itself. The disability is therefore the conditioning factor for physical activity. The most important point could be the subjective perception. Physical activity is often connected to exercise including endurance training (running/walking) or weight training. Physical movements where the heartbeat rises are often not perceived as physical activity or exercise, rather than everyday movement. Probably it needs more enlightenment in relation to physical activity and sports exercise. Nevertheless we believe it is important to establish studies that consider the individual subjective perception related to physical activity and the individual physical possibilities in pwMS. In terms of fatigue, excluding pwMS with a PDDS 3-8 seems not to have an impact on GLTEQ-HCT. There is still a significant inverse correlation between the physical activity level and fatigue (*p* = 0.008). More longitudinal and interventional studies remain essential to conclude whether changes in physical activity behaviour provoke changes in disease activity and/or progression. Additionally, mechanistically supported investigations can deepen the knowledge concerning the causality within these relationships. Furthermore a prospective randomized and controlled trial should be carried out over the time comparing those who undergo physical activity in the placebo group.

Finally, the restriction imposed by the COVID-19 pandemic might potentially have influenced participants´ physical activity assessment. Especially at the beginning of the COVID-19 pandemic, there was a major uncertainty about dealing with the disease. To analyze the effect in self-reported physical activity levels between pwMS recruited before versus after March 2020, where the first Covid-related lockdown in Germany took place. There is no significant difference in the self-reported physical activity level between pwMS who conducted the survey before the Corona-related lockdown in Germany in March 2020 and pwMS who conducted the survey after March 2020 (unpaired *t*-test: *p* = 0.485). The corona-pandemic situation seems not to have an impact on the physical activity levels in pwMS during the first months of the government ordered lockdown.

## 5. Conclusion

Our results suggest that self-reported physical activity is related to disease severity and fatigue in pwMS. However, self-reported physical activity does not significantly affect the annualised relapse rate. By excluding non-ambulatory pwMS there is no significant relation between physical activity level and disease severity. The physical disability could be the conditioning factor for self-reported physical activity. To assess whether changes in physical activity behaviour lead to changes in disease activity, more high evidence quality interventional studies are needed.

## Data availability statement

The datasets presented in this study can be found in online repositories: https://github.com/MaritSchlagheck/MS-survey. Any further enquiries can be directed to the corresponding author.

## Author contributions

MLS: conceptualization, methodology, formal analysis, writing - original draft. SH: methodology, writing - original draft. NJ, DW, AR, and FW: conceptualization, methodology, writing - review & editing. JB: conceptualization, resources, writing - review & editing. CW: conceptualization, writing - review & editing. PZ: conceptualization, methodology, supervision, writing - review & editing. All authors contributed to the article and approved the submitted version.

## Conflict of interest

The authors declare that the research was conducted in the absence of any commercial or financial relationships that could be construed as a potential conflict of interest.

## Publisher’s note

All claims expressed in this article are solely those of the authors and do not necessarily represent those of their affiliated organizations, or those of the publisher, the editors and the reviewers. Any product that may be evaluated in this article, or claim that may be made by its manufacturer, is not guaranteed or endorsed by the publisher.
